# Inorganic Nanoparticles: Toxic Effects, Mechanisms of Cytotoxicity and Phytochemical Interactions

**DOI:** 10.34172/apb.2022.077

**Published:** 2021-10-06

**Authors:** Rashid Bhatti, Hadia Shakeel, Kausar Malik, Muhammad Qasim, Mohsin Ahmad Khan, Nadeem Ahmed, Shajia Jabeen

**Affiliations:** ^1^Molecular Medicine Lab, Centre of Excellence in Molecular Biology, University of the Punjab, Lahore, Pakistan.; ^2^Department of Bioinformatics and Biotechnology, Government College University, Faisalabad, Pakistan.; ^3^Development of Recombinant Biopharmaceuticals Lab, Centre of Excellence in Molecular Biology, University of the Punjab, Lahore, Pakistan.

**Keywords:** Medicine, Nanoparticles, Phytochemicals, Toxicity, Therapeutic effects

## Abstract

During the last few decades, nanotechnology has gained many applications in almost all fields of life because of the unique properties of nanoparticles (NPs). Nanotechnology has specially marked its name in the field of medicine. However, NPs toxicity is detrimental to human health and is a prime concern in applied medicine. They can cause insomnia, vertigo, madarosis, epistaxis, hypokalemia, lymphopenia, Alzheimer’s and Parkinson’s diseases, etc. There is a gap in knowledge regarding the study of the toxicological effects of NPs. Mechanisms that are responsible for this toxicity are not fully understood yet. Phytochemicals have natural therapeutic effects of reducing metal NPs’ toxicity by acting as stabilizers and nontoxic reducing agents. However, the interaction between phytochemicals and NPs is remained to be elucidated. This review will provide in-depth knowledge about the various types of inorganic NPs and their associated toxicities, key parameters determining the toxic behaviour of NPs, and the mechanisms behind their cytotoxicity. It also emphasizes the need for further research to understand the interaction between various phytochemicals and NPs for therapeutic purposes.

## Introduction

A lot of progress has been made in the area of nanotechnology over the last few decades. Nanoparticles (NPs) usually have a nano scale size, i.e., a diameter of less than or equal to 100 nm.^1^ Because of their unique properties, they have applications in numerous fields including cosmetics, electronics and medicine.^2^ Silver, gold, zinc oxide (ZnO), and titanium oxide (TiO_2_) NPs are used in cosmetics because of their excellent drug delivery system, skin whitening and moisture retention properties. Their use in cosmetics is safe as they do not penetrate the skin. Therefore, they are not as harmful as long as they are used dermally.^3^ NPs use in diagnostics and therapeutics is growing day by day. However, safety is needed to be ensured for their effective use in various fields like food, cosmetics, medicine, etc.^4^ Plants possess abundant radical scavenging molecules like vitamins, phenolic compounds, terpenoids, etc. These molecules have antioxidant activity, thus enabling them to reduce toxicity caused by NPs.^5^ As NPs are toxic to health, workers dealing with them must wear personal protective equipment (PPE) such as respirators, nitrile gloves, lab coats, goggles and closed-toed shoes. Fume hoods, gloves, biosafety cabinets should be employed for handling NPs.^6^

 This review discusses different types of iNPs, their toxicity, factors affecting toxicity of iNPs, mechanisms behind their toxicity, strategies to avoid toxicity and the interaction between phytochemicals and inorganic NPs.

## Types of inorganic nanoparticles and their toxicity

Of all the NPs, inorganic NPs (quantum dots, metallic NPs, etc) are among those that are most abundantly produced and used commercially.^7^ They are being used as therapeutic agents because of their anticancer and antimicrobial activities.^8^ They can be used to create antimicrobial nanocomposite films. TYiO2-NPs were incorporated into chitosan to produce a biocomposite membrane that reduced the oxidative stress levels and apoptosis in mouse fibroblast cells due to the superior porosity, crystallinity, mechanical strength and structural flexibility.^9^

However, their increased exposure may cause inflammation, genotoxicity, and oxidative stress, leading to cancer and metabolic diseases.^10^ Different types of inorganic NPs and their associated toxicities are mentioned in Table 1.

**Table 1:** Inorganic nanoparticles and their toxicity

**Type of inorganic nanoparticle**	**Source reducing Agent**	**Particle size**	**Mechanism**	**References**
TMAT-AuNP	Gold	1.3 nm	Progression of eye pigmentation	^11^
Ag-NP	Silver	10 nm	Oxidative stress	^12^
Multi-walled carbon nanotubes	Carbon nanotubes	15-50 nm	Inflammation	^13^
TiO_2_	Titanium	5-90 nm	Apoptosis	^14^
ZnS CdS Quantum dots (QD)	Cores: Zinc Cadmium Shells: Sulphide	10 ± 2 nm 8 ± 2 nm	Increased lipid peroxidation & catalase activity	^15^

###  Gold and silver nanoparticles toxicity

“Nanogold” is a suspension of sub-micrometer-sized gold particles in a fluid, usually water.^16^ Because of chemical stability and good optical properties, gold nanoparticles (AuNPs) are being used in chemotherapy and drug delivery. They have shown the cytotoxicity *in vitro* on BALB/3T3 mouse fibroblasts.^17^

Silver nanoparticles (AgNPs), because of their antimicrobial activity, are used in medicine and drug delivery.^18^ Oxidation of AgNPs results in the release of silver ions that accounts for cytotoxicity related to the AgNPs.^19^ A study showed that reactive oxygen species (ROS) generation was more by AgNPs than bulk silver, due to which AgNPs are more toxic than bulk silver.^20^ Actually, the toxicity of AgNPs is related to surface area; as the concentration of AgNPs per unit volume of reaction mixture increases, the surface area increases as well. It causes an increase in ROS production, which ultimately contributes to cell toxicity.^21^ Moreover, oxidative damage and subacute toxicity of AgNP-PVP and AgNP-20 on the kidneys, lungs and liver of mice have also been reported.^22^

###  Carbon nanotubes toxicity

Carbon nanotubes (CNTs) are the allotropes of carbon and possess fiber-shaped nanostructures.^23^ In cell lines, CNTs can activate ROS-associated intracellular signalling pathways.^24^ They have also been reported to trigger the release of cytokines including TNF-α, IL-1β, IL-8 and IL-6 from macrophages and mesothelial cells.^25^ A study showed that nanocomposites of chitosan CNTs not only improved antimicrobial activity but also caused DNA damage in hepatic cells of *Oreochromis niloticus.*^26^

###  Titanium dioxide nanoparticles toxicity

Titanium dioxide nanoparticles (TiO_2_ NPs) are used in cosmetics, food additives and pharmaceutical products because of their chemical stability and photocatalytic properties. TiO_2_ NPs can induce cytotoxicity, genotoxicity and oxidative stress.^27^ They have induced indirect genotoxicity in two lung cell lines, i.e., A549 and BEAS-2B due to impaired DNA repair processes.^28^

###  Quantum dots nanoparticles toxicity

Quantum dots (QDs), the semiconductor NPs, are fluorescent and possess unique optical properties.^29^ Just like other NPs, QDs cytotoxicity depends on their shape, size, concentration, redox activity, mechanical stability, surface coatings and charge.^30^ Nitrogen and Sulphur co-doped graphene QDs are less toxic and used as fluorescent nano-sensors in living cells.^31^

## Factors affecting the toxicity of NPs

 Major factors associated with NPs toxicity are given below:

###  Dose and time of exposure

The toxicity of NPs is associated with their number. Cells with more particles have more toxic effects than cells with fewer particles. Both dose and time play a crucial role in determining the toxicity of NPs.^32^ However, NP penetration in the cells depends on their exposure time.

###  Concentration and aggregation

Increased concentration of NPs favours their aggregation as their size is in micrometers, they do not penetrate the cells and their toxicity is lost.^33^ On the other hand, another study suggested that aggregation of NPs affects their stability, making them more toxic.^34^

###  Particle size and shape

The toxicity of NPs also depends on their size.^35^ Small-sized NPs are toxic than large-sized NPs, e.g., AgNPs of 10 nm have more significant toxicity than the larger AgNPs (20-100 nm).^36^ The shape is another factor that helps to determine the toxicity of NPs, i.e., different aspect ratios possess different toxicity levels.^37^ Long asbestos fibers (10 µm) can cause lung cancer, while short fibers (5-10µm) can cause mesothelioma or asbestosis (2 µm).^38^ Multi-walled CNTs embedded in pleural membrane activated macrophages that secreted IL-1β, which amplify inflammation in mesothelial cells.^39^

###  Crystal structure and route of exposure

Different crystalline structures of NPs can exhibit toxicity differently. NPs can show different oxidative mechanisms, cellular uptake and subcellular localization based upon their crystalline structure.^33^ Route of exposure regulates the initial interaction of NPs and cells.^40^ Dermal exposure of NPs activate the immune system while their systemic distribution causes spleen and liver toxicities.^36^

###  Pre-exposure and surface functionalization

Pre-exposure to low nanoparticle concentrations can stimulate phagocytic activity and adapt the human body to these NPs.^41^ Whereastheir surface properties have drastic effects on oxidation processes. NPs with cationic surface are more cytotoxic than NPs with anionic surface.^42^

## Mechanisms of nanoparticles cytotoxicity

**Figure 1:**
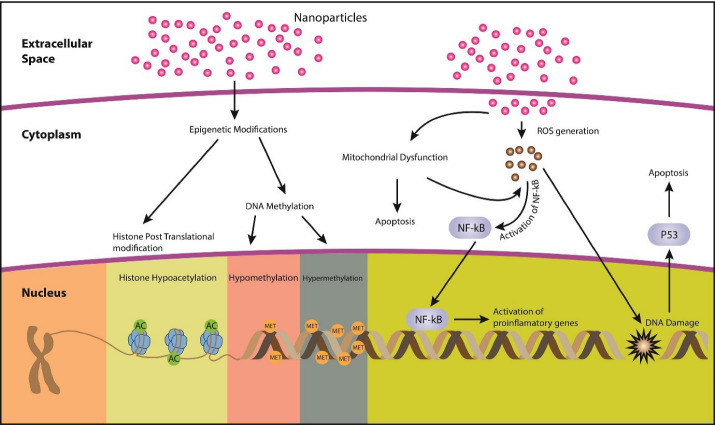
Different mechanisms associated with nanoparticle toxicity

Nanoparticle toxicity mechanisms include DNA damage, oxidative stress, ROS production (Figure 1), and alteration of protein structures. Different mechanisms associated with nanoparticle cytotoxicity are mentioned in Table 2.

**Table 2:** Inorganic nanoparticles and their cytotoxicity

**Type of inorganic nanoparticle**	**Source reducing agent**	**Particle size**	**Mechanism of cytotoxicity**	**References**
Au-NP	Gold	25-30 nm	Oxidative stress	^43^
Ag20Pep	Silver	20 nm	ROS formation & calcium dysregulation	^44^
Long and short multi-walled carbon nanotubes	Carbon	7-26 nm	Lipid peroxidation and oxidative stress	^45^
TiO_2_	Titanium	~26.4 ± 1.2 nm	ROS production and apoptosis	^46^
CdTe quantum dots	Cadmium	2.3 nm	ROS generation and apoptosis	^47^

 Two important cytotoxicity mechanisms are discussed below:

###  Inflammatory response and Oxidative stress

Inflammation is the defense mechanism of the body that involves many cytokines.^48^ Macrophages in the macrophage-rich organs, including spleen and liver, usually take up the NPs and release cytokines.^49^ ROS induction is the leading cause of nanotoxicity.^50^ A large number of nanomaterials have induced toxicity in human erythrocytes and skin fibroblasts through the production of ROS.^51^ Moreover, an imbalance in redox state of the cell causes the oxidative stress.^52^ Though ROS production is considered normal, but its excessive production is harmful to the cells. ZnO-NPs increase ROS inside cells and activate apoptosis via the caspase cascades in human gingival squamous cell carcinoma.^53^

###  Epigenetic modifications

Epigenetic modifications refer to the heritable changes that are not due to alterations in the nucleotide sequence of DNA. Instead, they are due to the alterations in chromatin structure and DNA accessibility, e.g., histone modification and DNA methylation.^54^ Transcriptional machinery of the cell depends upon how tightly DNA is enfolded around histones, while DNA packaging depends upon histone post-translational modifications.^55^ Nanoparticle exposure can lead to epigenetic changes. Inorganic nanoparticles (iNPs) can change the gene and chromatin packaging, e.g., Ag-NPs can cross the nuclear membrane and interfere with chromatin remolding enzymes that affect condensation of chromatin and accessibility of DNA, thus altering the expression of genes.^56^

## Strategies to avoid toxicity caused by inorganic nanoparticles

The main cause of iNPs toxicity is oxidative stress, so their toxicity can be overcome by preventing oxidative stress. Interestingly, it is reported that vitamin C can decrease ROS production in acute myeloid leukaemia cells treated with AgNPs.^57^ Another strategy that can be used to avoid oxidative stress is to use methods that slow down the release of metal ions since metal ions play a role in the induction of oxidative stress, e.g., slowing down the release of silver ions produced by AgNPs can reduce AgNP-induced toxicity.^58,59^ NPs can be coated with antioxidants or a polymer like polyethylene glycol (PEG) to reduce ROS formation. PEG-coated iron oxide NPs reduce cytotoxicity by blocking the interaction of ROS with Fe_2_O_3_-NPs.^60^ PVP-Bi2Se3 NPs showed better radiotherapy efficacy in cancer treatment. As selenium can improve immune function by reducing the harmful effects of radiation on normal cells.^61^ NPs toxicity can also be minimized by creating metal oxide NPs that are toxic to cancer cells but not to normal cells, e.g., ZnO NPs selectively target cancerous cells leaving normal cells.^62^

## Interaction between phytochemicals and inorganic nanoparticles

Secondary metabolites derived from harmless microbes and plants are called phytochemicals.^63^ These phytochemicals due to their therapeutic effects are used to prepare metal NPs by green synthesis approach. Green synthesis is a biological method for synthesizing NPs based upon oxidation-reduction reaction to reduce metal ions into stable NPs using an organism’s components or its extract.^64^ Previous studies showed that phytochemicals with antioxidant properties could possess the ability to protect cells from NPs’ exposure. However, the interaction between phytochemicals, NPs, and their associated toxicities are yet to be understood.

## Conclusion

 NPs are being used in almost all fields of life today, but nanotoxicity has become a major issue. Oxidative stress is particularly associated with the toxicity of inorganic NPs and reducing this stress may increase the biocompatibility of NPs. Due to low toxicity and high bioactivity, phytochemicals can be coated on NPs to reduce their cytotoxicity efficiently. Research on the toxicity of iNPs is highly dispersed and no definitive conclusions can be drawn from the available literature. So, there is a need for further research to understand the toxicity mechanisms, the interaction between various phytochemicals and inorganic NPs and investigate strategies for synthesizing NPs with optimal properties while minimizing adverse effects on living cells.

## Acknowledgments

 The authors would like to thank Ms. Zainab Akram and Ms. Khadija Abdul Majid for the help rendered by them.

## Ethical Issues

 Not applicable.

## Conflict of Interest

 The authors declare that they have no conflict of interest.
